# Use of veno-pulmoarterial ECMO to manage differential oxygenation in a lung transplant candidate

**DOI:** 10.1177/02676591251394852

**Published:** 2025-11-03

**Authors:** Ryo Fujimoto, Asad Usman, Salim Olia, Audrey Spelde, Paulo Gregorio, Zane Mazur, Thomas Richards, Maria Crespo, Christian A. Bermudez

**Affiliations:** 1 Department of Surgery, Division of Cardiovascular Surgery, 21798 University of Pennsylvania, Philadelphia, PA, USA; 2 Department of Anesthesiology and Critical Care, 21798 University of Pennsylvania, Philadelphia, PA, USA; 3 Department of Pulmonary, Allergy and Critical Care, 21798 University of Pennsylvania, Philadelphia, PA, USA

**Keywords:** extracorporeal membrane oxygenation, veno-pulmoarterial, veno-arterial, differential oxygenation, lung transplantation

## Abstract

A 44-year-old male with pulmonary sarcoidosis related interstitial lung disease and pulmonary hypertension was admitted for pre-transplant evaluation. During hospitalization, he developed cardiac arrest due to worsening respiratory failure and was immediately placed on veno-arterial (VA) extracorporeal membrane oxygenation (ECMO). Despite initial improvement, he developed differential oxygenation, which was managed by converting the support to veno-venoarterial (VVA) ECMO. However, pulmonary artery pressure increased, placing a greater load on the right ventricle, and oxygenation remained inadequate, leading to further optimization of the ECMO settings. The support was switched to veno-pulmoarterial (VPA) ECMO, which improved oxygenation and, through the combined effects of oxygenation and circulatory support, stabilized his condition. After 24 days of ECMO support, the patient underwent successful lung transplantation. His postoperative course was uncomplicated, and he was discharged on postoperative day 32. Over the subsequent 2 years of follow-up, he has remained active, independent of oxygen, and free of functional limitations.

## Introduction

Extracorporeal membrane oxygenation (ECMO) is an established bridge to lung transplantation, with veno-arterial (VA) ECMO providing cardiorespiratory support but carrying risks of complications.^1^ For adults supported by peripheral VA ECMO, retrograde flow from the femoral artery mixes with native output in the aorta; when left ventricular function recovers in severe respiratory failure, this may cause differential oxygenation with poorly oxygenated blood perfusing the upper body, including the heart and brain.^2–4^ This report describes a lung transplant candidate who developed differential oxygenation while supported on VA ECMO, prompting conversion to veno-pulmoarterial (VPA) ECMO, which enabled successful lung transplantation.

## Case report

The patient was a 44-year-old male who was admitted for pre-transplant evaluation due to pulmonary sarcoidosis related interstitial lung disease and pulmonary hypertension, requiring nasal high-flow oxygen therapy at 45 L/min. On day 10 of hospitalization, due to worsening respiratory status and cardiac arrest, he underwent emergency intubation and was placed on bifemoral VA ECMO, with 25 Fr Medtronic multistage drainage cannula (60 cm length) in the venous position and a 16 Fr OptiSite single-stage arterial return cannula (15 cm length) (Figure 1(A)). Transthoracic echocardiography found severely impaired biventricular function with ejection fraction (EF) 15 %. On day 4 of ECMO support, despite achieving a VA ECMO flow of 5.06 L/min with an FdO2 of 1.0, and inhaled nitric oxide, oxygenation remained inadequate, the right radial arterial PaO2 level dropped to 59 mmHg under ventilator settings of FiO2: 1.0, PEEP: 11.5 cm H2O.The simultaneous decrease in right radial PaO2, neurological deterioration, with the Glasgow Coma Scale (GCS) declining from 10 to 3, and LVEF improvement to 25% shown on a transesophageal echocardiogram (TEE) led to suspicion of differential oxygenation. To manage differential hypoxia, the support was converted to veno-venoarterial (VVA) ECMO with additional ECMO return 15 Fr Maquet HLS single-stage return cannula (15 cm length) inserted via the right internal jugular vein (RIJ) into the superior vena cava (SVC) near the cavoatrial junction (Figure 1(B)). After the conversion, while oxygenation slightly improved with a PaO2/FiO2 ratio of 90–110 mmHg, the pulmonary artery pressure subsequently increased from 51/22 mmHg to a range of 86/40 to 110/55 mmHg. On day 17 of ECMO support, however, despite optimization of ECMO settings (flow: 5.14 L/min (RIJ 3.1 L/min), FdO2: 1.0), right radial arterial blood gas (ABG) analysis indicated PaO2: 71 mmHg under FiO2 1.0 and PEEP 11.5 cm H2O, while the patient was receiving vasopressin (0.26 mU/kg/min, vasopressor) and milrinone (0.3 μg/kg/min, inodilator). TEE showed severe right ventricular dysfunction, improved left ventricular EF (35%), and severe tricuspid regurgitation. Considering these findings, the patient was transitioned to a veno-pulmonary (VP) ECMO. A 19 Fr Medtronic single-stage venous return cannula (50 cm length) was advanced from the RIJ to the main pulmonary artery, establishing a VP ECMO circuit with venous drainage via the right femoral vein and reinfusion into the pulmonary artery. Additionally, incorporation of the left femoral arterial cannula into the arterial limb of the circuit using a Y-connector established VPA ECMO, with a Hoffman clamp applied to regulate flow distribution, thereby diverting part of the pulmonary arterial flow to the systemic circulation to prevent excessive pressure on the pulmonary artery, while allowing oxygenated blood to be supplied to the aortic root (Figure 1(C)). VP ECMO flow was maintained at 2–3 L/min. Immediately following the conversion, right radial arterial oxygenation improved significantly, with a PaO_2_/FiO_2_ ratio of 452 mmHg. Concurrently, pulmonary artery pressures decreased to 66/18 mmHg. His condition subsequently stabilized as his GCS improved from 3 to 9, and he underwent lung transplantation after 24 days of ECMO support. He had an uneventful recovery without ECMO and was discharged on postoperative day 32. He has been followed for 2 years, remaining ambulatory without oxygen and without limitations in daily activities.

**Figure 1.**
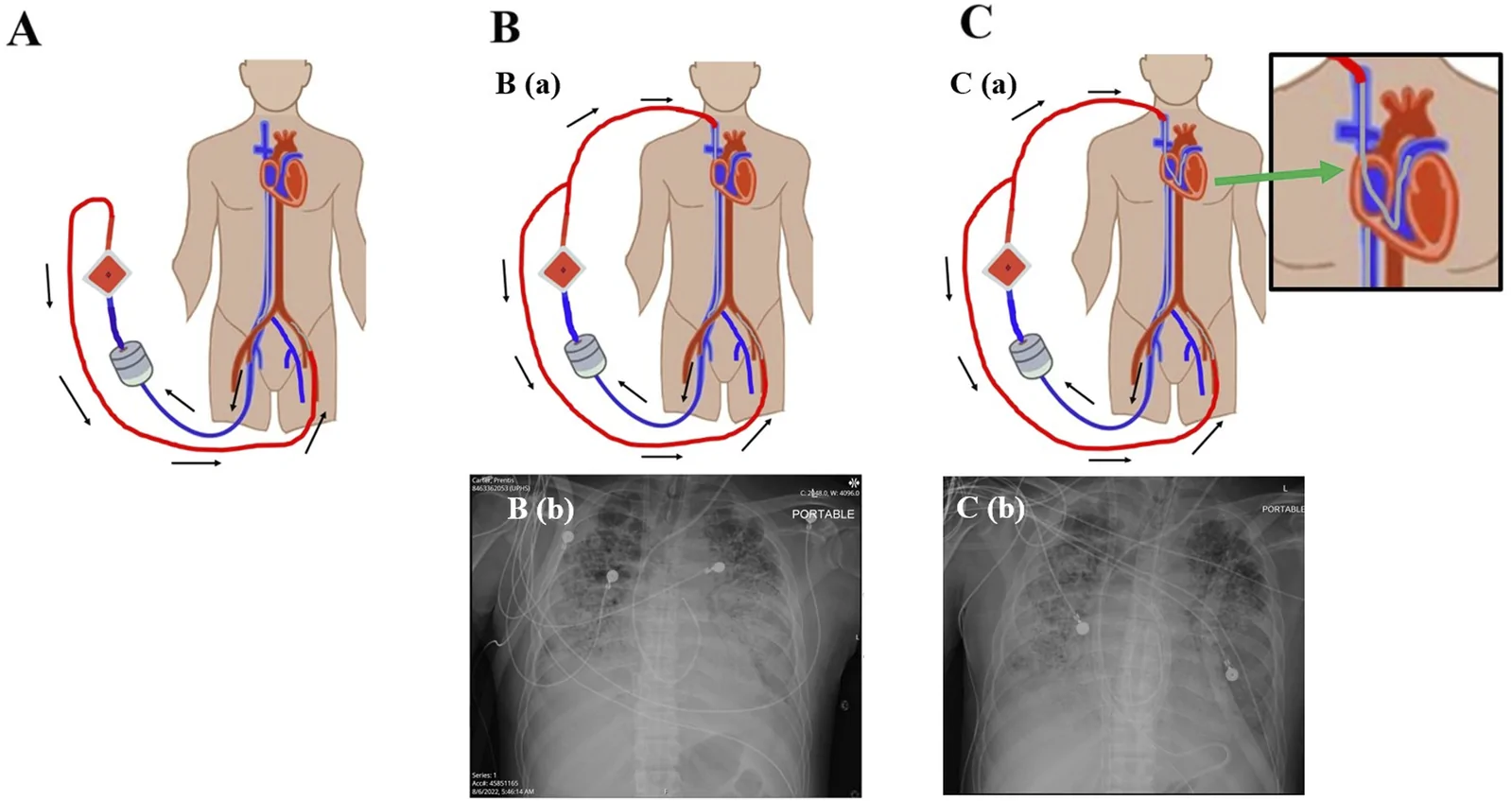
(A) Bifemoral veno-arterial extracorporeal membrane oxygenation (VA ECMO) with venous drainage via the femoral vein and retrograde arterial return through the contralateral femoral artery. (B) (a) Schematic illustration of veno-venoarterial extracorporeal membrane oxygenation (VVA ECMO). This configuration consists of single venous drainage from the right femoral vein and arterial return through the left femoral artery and superior vena cava (SVC). (b) Chest radiograph demonstrating the additional ECMO return cannula inserted via the right internal jugular vein (RIJ) and positioned in the SVC, with a drainage cannula placed in the inferior vena cava (IVC) via the femoral vein. (C) (a) Schematic illustration of veno-pulmoarterial extracorporeal membrane oxygenation (VPA ECMO). This configuration consists of single venous drainage from the right femoral vein and arterial return through the left femoral artery and the pulmonary artery. (b) Chest radiograph showing a 19 Fr Medtronic Bio-Medicus single-stage venous cannula advanced from the RIJ into the main pulmonary artery, with a drainage cannula placed in the IVC via the femoral vein.

## Discussion

Many lung transplantation candidates necessitate invasive mechanical ventilation and ECMO support while on the waiting list.^1^ In this case, acute respiratory failure caused hypoxemia that led to cardiogenic shock and subsequent cardiac arrest, requiring VA-ECMO as a bridge to transplantation. Differential oxygenation arises when desaturated blood from the pulmonary circulation is ejected by the left ventricle and mixes with oxygenated blood delivered via the ECMO arterial cannula.^2–4^ In this case, after the insertion of VA ECMO, left heart function improved, and subsequently, a decrease in radial artery oxygen partial pressure and a decline in the level of consciousness were observed, pointing to the development of differential oxygenation. While in cases of differential oxygenation, non-invasive parameters are first optimized,^5–7^ modifying or adding to the ECMO circuit may resolve the condition; for example, inserting a drainage cannula via the RIJ with its tip in the SVC can prevent desaturated blood from being ejected from the left ventricle without mixing with oxygenated ECMO blood.^8^ In this case, as differential oxygenation persisted, a second return cannula was inserted via RIJ to convert to VVA ECMO, delivering oxygenated blood to the right atrium and improving pulmonary artery oxygenation.^2–4^ Percutaneous VP ECMO has recently emerged for progressive RV failure by delivering oxygenated blood into the pulmonary artery.^9^ In this patient, switching to VPA ECMO (hybrid of VP and VA, i.e. return to both the pulmonary artery and the systemic arterial side) improved the condition. However, VP ECMO may cause progressive pulmonary edema and hinder lung recovery.^9^ To prevent this complication, we carefully adjusted VP component flow to avoid excessive flow, maintaining it at 2–3 L/min, which allowed for adequate oxygenation to be sustained and successfully bridged the patient to lung transplantation (Table 1).

**Table 1.** Blood gases (right radial artery) and laboratory tests from hospital admission to pre-transplant period under ECMO support.

	Hospital admission	Before VA ECMO	One day after VA ECMO	Before VVA ECMO	One day after VVA ECMO	Before VPA ECMO	One day after VPA ECMO	Before LTx
pH	7.43	7.37	7.41	7.52	7.52	7.49	7.50	7.33
PaO2 (mmHg)	NA	46	204	59	115	71	452	322
PaCO2 (mmHg)	50	46	47	42	36	47	34	54
Lactic acid (mmol/L)	1.1	3.7	4.4	1.2	0.9	1.2	0.9	0.7
Creatinine (mg/dl)	0.9	1.10	0.98	0.86	0.69	0.75	0.74	0.44
Total bilirubin (mg/dl)	0.4	1.3	1.5	0.7	1.2	1.1	1.6	1.4

NA, not available; VA ECMO, veno-arterial extracorporeal membrane oxygenation; VVA ECMO, veno-venoarterial extracorporeal membrane oxygenation; VPA ECMO, veno-pulmoarterial extracorporeal membrane oxygenation; LTx, lung transplantation.

## Conclusions

In a lung transplantation candidate, VPA ECMO (hybrid of VP and VA) provided effective management of differential oxygenation and right ventricular failure, with the former persisting despite conversion to VVA ECMO, thereby facilitating a successful bridge to lung transplantation.

## Data Availability

Data supporting the findings of this study are available from the corresponding author upon reasonable request.*
